# Volatile Organic Compounds Sensing Using Optical Fibre Long Period Grating with Mesoporous Nano-Scale Coating

**DOI:** 10.3390/s17020205

**Published:** 2017-02-08

**Authors:** Jiri Hromadka, Sergiy Korposh, Matthew Partridge, Stephen W. James, Frank Davis, Derrick Crump, Ralph P. Tatam

**Affiliations:** 1 Optics and Photonics Group, Faculty of Engineering, University of Nottingham, University Park, Nottingham NG7 2RD, UK; Jiri.Hromadka@nottingham.ac.uk; 2 Institute for Environmental Studies, Charles University in Prague, Benatska 2, Prague 2 CZ-128 01, Czech Republic; 3 Centre for Engineering Photonics, Cranfield University, Cranfield, Bedfordshire MK43 0AL, UK; m.c.partridge@cranfield.ac.uk (M.P.); s.w.james@cranfield.ac.uk (S.W.J.); r.p.tatam@cranfield.ac.uk (R.P.T.); 4 Department of Engineering and Applied Design, University of Chichester Bognor Regis, West Sussex PO21 1HR, UK; F.Davis@chi.ac.uk; 5 Environmental Science and Technology Department, Cranfield University, Cranfield, Bedfordshire MK43 0AL, UK; d.crump@cranfield.ac.uk

**Keywords:** long period grating (LPG), volatile organic compounds (VOCs), phase matching turning point (PMTP), mesoporous film, layer-by-layer (LbL), p-sulphanato calix[8]arene (CA[8]), p-sulphanato calix[4]arene (CA[4])

## Abstract

A long period grating (LPG) modified with a mesoporous film infused with a calixarene as a functional compound was employed for the detection of individual volatile organic compounds (VOCs) and their mixtures. The mesoporous film consisted of an inorganic part, SiO_2_ nanoparticles (NPs), along with an organic moiety of poly(allylamine hydrochloride) polycation PAH, which was finally infused with the functional compound, p-sulphanato calix[4]arene (CA[4]) or p-sulphanato calix[8]arene (CA[8]). The LPG sensor was designed to operate at the phase matching turning point to provide the highest sensitivity. The sensing mechanism is based on the measurement of the refractive index (RI) change induced by a complex of the VOCs with calixarene. The LPG, modified with a coating of 5 cycles of (SiO_2_ NPs/PAH) and infused with CA[4] or CA[8], was exposed to chloroform, benzene, toluene and acetone vapours. The British Standards test of the VOCs emissions from material (BS EN ISO 16000-9:2006) was used to test the LPG sensor performance.

## 1. Introduction

Most people spend more than 90% of their time indoors [1], in daily contact with household products, such as paints and paint strippers, solvents, sprays and wood preservatives, disinfection products, repellents and air fresheners, all emitting or containing volatile organic compounds (VOCs). VOCs consist of a broad mixture of gases and cause various short-term and long-term (delayed) adverse health effects, with some proven as animal or human carcinogens. They are also determined as one of the possible causes of sick building syndrome (SBS) [2].

The indoor concentration of VOCs is higher in comparison to outdoor levels [2]. The mean total VOCs (TVOCs) concentration was measured to be 0.2–0.4 mg·m^−3^ during a survey of British households [3].

There are guidelines for individual substances (e.g., formaldehyde, benzene) set by the World Health Organization (WHO) for homes [4], while the occupational limits are set by the Occupational Safety and Health Administration (OSHA) in the US [5] and by the Health and Safety Executive (HSE) in UK [6]. Scientific evidence shows that the irritation of the occupants is associated with indoor concentration of TVOCs in the range 0.2 to 5 mg·m^−3^ [3], while no effect seems to be observed at concentrations below 0.2 mg·m^−3^ [7].

As material leakage represents the most significant indoor source of VOCs (50%, the remainder is associated with human activities), the overall acceptable emission rate of the materials should be kept below 0.03 mg·m^−2^·h^−2^ (the approximation considers the size of the room, possible leakage surface area and ventilation rate) [2].

The legal framework concerning the testing of VOCs leakage from paints is covered by EU 2004/42/CE, which sets the limits for VOCs emissions that occur due to use of organic solvents in certain paints, varnishes and vehicle refinishing products. The guidelines for material testing are also covered by ISO standards, where following are the most appropriate EN ISO 16000-9—Indoor air—Part 9—Determination of the emission of volatile organic compounds from building products and furnishing—Emission test chamber method and EN ISO 16000-10 Determination of the emission of volatile organic compounds from building products and furnishing—Emission test cell method).

Passive flux sampling, emission test cells (ETC) and field and laboratory emission cells (FLEC) have been considered to be useful for material leakage measurements to determine the level of VOCs [8]. Gas chromatography-mass spectroscopy (GC-MS) were used for VOC analysis in this case [8].

Passive sampling does not require an air pump and an air-flow meter, but very volatile agents can be lost because of back-diffusion [9]. Passive samplers provide average concentration data for an exposure period that is often days or weeks to achieve appropriate sensitivity. Passive badges use charcoal or other media as an adsorbent. The badge is left in the environment during the sampling period (commonly from 8 h to 1 week) and then is sent to the lab for the further analysis [10]. When measurement of the concentration dynamic (changes of exposure in time) is required, an active (pumped) approach is applied [11]. Real time measurement is undertaken using photo-ionization detectors (PIDs), but the accuracy depends on the VOCs present in the actual mixture [10]. While longer time averages may be more relevant when possible health effects of the pollutant are considered, in the case of chronic effects the concentration dynamic is important [9].

The most precise and also the most expensive approach is active sorption/chemical analysis. The sampled air is flowed by the pumps into the tubes which include the sorbent of organic polymer resins (e.g., Tenax) or activated charcoal. The sorbent traps the present VOCs and GC-MS is then used for VOCs analysis. The VOCs are measured individually and then the TVOCs is subsequently calculated [10].

ETCs and FLECs can be small and portable or can be large scale, with volumes of up to 80,000 dm^3^. The chambers are usually fabricated from glass or stainless steel, because there should be no reaction between the surface of the chamber and the analyte of interest. The condition inside the cells can be controlled and FLECs have the advantage that can be used outside the lab. The air flows through the cell to the sorbent tubes and the trapped VOCs are further analysed by GC-MS [8]. While large-scale ETCs allow the testing of complete, intact equipment, the experiment takes a long time and is highly expensive. The costs are smaller for FLECs, with passive flux samplers being less expensive still [8]. Application of the inexpensive and portable sensors with sufficient sensitivity that can provide results in real time to measure leakage of VOCs is highly desirable and will benefit both industry and consumers.

Fibre optic-based TVOCs sensors could potentially provide real time measurements, sufficient accuracy and low cost. Other advantages include their small size, they can work even in extreme conditions, enable remote monitoring with no electrical power needed at the sensing point and in addition they are immune to electromagnetic interference [12].

A variety of fibre optic based chemical sensing platforms have been reported and investigated during the last decade [13,14]. Among them, fibre optic long period gratings (LPGs) modified with appropriate functional materials have been considered to provide high sensitivity and selectivity towards target compounds [15,16,17,18,19,20,21,22].

An LPG consists of a periodic perturbation of the refractive index of the optical fibre core, which couples fundamental core mode into the co-propagating cladding modes. This coupling is manifested in the transmission spectrum of the optical fibre as a series of loss, or resonance bands. Each of them corresponds to coupling to a different cladding mode and each shows a different sensitivity to environmental perturbation [23]. 

The wavelengths at which the core mode is coupled to cladding modes are determined by the phase matching Equation (1):

(1) $$\lambda_{x}=\left(n_{core}-\;n_{clad\left(x\right)}\right)\Lambda$$

where *λ_x_* represents the wavelength at which light is coupled to the LP_0x_ cladding mode, *n_core_* is the effective refractive index of the mode propagating in the core of the fibre, *n_clad(x)_* is the effective index of the LP_0x_ cladding mode and *Λ* is the period of the LPG [23].

The sensitivity of LPGs to external perturbations such as strain, temperature or refractive index (RI) change relies on the measurement of the shifts of the central wavelengths of the resonance bands that are proportional to the magnitude of the measurand [23]. 

For the development of fibre optic chemical and bio-sensors, the surface of the optical fibre is modified with a functional material that can selectively interact with the target compound. The selection of the material determines the analytes to which the coated LPG is sensitive and the thickness influences the sensitivity [24]. The response of the transmission spectrum of the LPG to the nanoscale coating deposition is characterized by a shift of the central wavelengths of the attenuation bands, which are caused by the change of the effective index of the cladding modes, and this change is optical-thickness dependent [25]. In addition, the highest sensitivity and the best performance are obtained when the LPG operates at or near the phase matching turning point (PMTP) [26], requiring an appropriate combination of grating period and optical thickness of the coating. (The optical thickness is derived from the geometrical thickness of the coating multiplied by the refractive index of the coating.)

Fibre optic sensors containing LPGs with functional coating have been used to measuring relative humidity [20], ammonia [15,27] or VOCs [28,29,30], and can also be used for a range of other materials, e.g., in bio-sensing [19,22,31]. Various methods have been employed for the deposition of the functional film onto the optical fibre [15,21,30,31,32,33]. Among these methods, the layer by layer (LbL) technique (also known as electrostatic self-assembly (ESA) technique), based on the deposition of the oppositely charged materials, allows control of film thickness with molecular precision. Using an LbL approach, the entire coating can be made from the sensitive material, which reacts with the compound of interest, leading to a change of its refractive index. Another option is that the sensitive material is infused into a mesoporous coating [15,16]. The sensitive material should fulfil the following criteria: have appropriate morphology, porosity, flexibility and thickness for the sensing selectivity, and matrix porosity for the speed of analyte diffusion [34]. A range of functional coatings—calixarene [28,29], zeolites [35] or diphenylsiloxane and titanium cross-linker have been used to modify LPG based fibre optic sensors to measure VOCs [36].

In this work, a single mode optical fibre containing a long period grating (LPG) was modified with a mesoporous thin film that was infused with calixarene molecules that acted as the functional compound for VOCs detection. Calixarene molecules contain a number of phenol or resorcinol aromatic rings connected together to a larger ring and the whole molecule looks like a bowl [37]. The analyte of the interest reacts with calixarene and becomes temporarily entrapped, however only weak interactions occur (no covalent bond is created) which enables easy liberation from the cavity and this effect causes the reversibility of the sensor. The sensitivity of the reaction depends on the size and shape of the molecule of interest and for this reason the specific reactions to different VOCs may be expected [30]. 

The use of calixarenes for the detection of VOCs has been reported previously. Ca[8] was shown to be sensitive to chloroform [38], benzene and toluene [30] and the responses of CA[4] exposure to chloroform and benzene [29] and toluene [39] have been explored.

The LPG was exposed to concentrations up to the saturated vapour concentrations of acetone, benzene, chloroform and toluene. The influence of two different sizes of calixarene molecules on the performance of the VOCs sensor was investigated and a comparison of their sensitivities was undertaken*.* The sensor was also used to measure mixtures of VOCs leaking from two different types of paints, with aim of assessing its performance in real life applications. In addition, the chemical composition of the VOCs was monitored by GC-MS.

The experiment performed simulated the conditions stated in the previously mentioned British—ISO standards.

## 2. Materials and Methods

### 2.1. Materials

PAH (*M*_w_: 75,000), NaOH 1 M aqueous solution, NH_3_, benzene, toluene, chloroform and acetone were purchased from Sigma-Aldrich. SiO_2_ NPs (SNOWTEX 20L) was synthesised Nissan Chemical. p-sulphanato calix[8]arene (CA[8]) and p-sulphanato calix[4]arene (CA[4]) 1 mM solution was synthetized in house [40]. All of the chemicals were of analytical grade reagents and were used without further purification. Deionized water (18.3 MΩ·cm) was obtained by reverse osmosis followed by ion exchange and filtration (Millipore, Direct-QTM, Nottingham, UK).

### 2.2. Sensor Fabrication

An LPG of length 40 mm with a grating period of 110.9 μm was fabricated in boron-germanium co-doped optical fibre (Fibercore PS750, Fibercore, Southampton, UK) with a cut-off wavelength of 670 nm in a point-by-point fashion, side-illuminating the optical fibre by the output from a frequency-quadrupled Nd:YAG laser, operating at 266 nm. 

The selected grating period facilitates coupling to a higher order cladding mode (LP_019_) at the PMTP, where its response to external refractive index is largest [41]. The use of optical fibre with a short cut-off wavelength ensures that resonance bands at their PMTP lie towards the red end of the visible spectrum, allowing the transmission spectrum of the LPG to be monitored using an inexpensive light source, halogen lamp, and a low-cost CCD spectrometer.

The transmission spectrum of the optical fibre was recorded by coupling the output from a tungsten-halogen lamp (Ocean Optics HL-2000, Ocean Optics, Oxford, UK) into the fibre, analysing the transmitted light using a fibre coupled CCD spectrometer (Ocean Optics HR4000, Ocean Optics, Oxford, UK). The grating period was selected such that the LPG operated at or near the phase matching turning point, which, for coupling to a particular cladding mode (in this case LP_019_), ensured optimized sensitivity.

**Figure 1:**
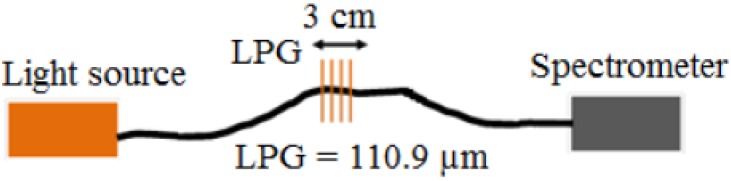
Schematic illustration of the long period grating (LPG) sensor.

The schematic illustration of the LPG sensor is shown in Figure 1.

### 2.3. Thin Film Deposition

A mesoporous thin film of SiO_2_ NPs was deposited onto the LPG using an electrostatic self-assembly approach as previously described [15,16,27]. An LPG was fixed in a Teflon holder constructed with a compartment to accommodate a solution. 

Briefly, the region of the optical fibre with the LPG was rinsed with deionized water and immersed in a 1 wt % NaOH ethanol and water (3:2, *v/v*) solution for 20 min, leading to a negatively charged surface. The optical fibre was then sequentially immersed into a solution containing a positively charged polymer, PAH, and a solution containing negatively charged SiO_2_ NPs for 20 min each, resulting in the alternate deposition of PAH and SiO_2_ NPs layers on the surface of the fibre. The fibre was rinsed with distilled water, and dried by flushing with nitrogen gas after each deposition step. The immersion in the PAH and SiO_2_ nanoparticles was repeated until the 5 layers were deposited. The transmission spectrum (TS) of the LPG was measured after each deposition. 

As was previously shown, functional compounds can be infused into mesoporous thin films [15] to provide sensor with its specificity to the desired analyte. CA[8] and CA[4] were used as the functional materials to provide the sensor reported here with the sensitivity to VOCs. In general, the thickness and porosity of the sensitive element affect the performance of coated LPG sensors [25,41]. It was previously shown that the optimal sensitivity to external refractive index was achieved [25] when 10 layers of SiO_2_ NPs thin film were deposited onto the surface of the LPG sensors. In this work, however, since the functional compound is infused into the mesoporous thin film, the optical thickness, the product of geometrical thickness and refractive index, changes, hence altering the optimal conditions of the LPG sensor. Here, 5 layers were found to provide the optimal performance of the LPG sensor in terms of sensitivity and response time.

The film porosity affects the refractive index of the film. The mesoporous structure created by silica nanoparticles enables the infusion of the sensitive element—calixarene. The porosity between the nanoparticles affects the amount of possible binding sites for the calixarene-VOC complexes and thus indicates the VOCs sensitivity. 

The film porosity affects the refractive index of the film, in this case allowing the deposition of a coating of refractive index 1.2 [15]. The deposition of a well-developed mesoporous structure of SiO_2_ nanoparticles film with a mean pore radius of 12.5 nm and specific surface area of 50 m^2^·g^−1^ onto an LPG was reported previously [15,42]. The mesoporous structure created by silica nanoparticles enables the infusion of the sensitive element—calixarene, which also acts to raise the refractive index of the coating. Calixarenes form a natural cavity with an approximate diameter of around 1–2 nm. These small cavities allow condensation of the target vapour below the natural condensation point of the vapour [39]. The pore dimensions influence the number of possible binding sites for the calixarene-VOC complexes and thus affect the sensitivity to VOCs.

The LPG modified with the (PAH/SiO_2_)_5_ coating was immersed into a 1 mM of CA[8] aqueous solution for 2 h followed by washing and drying. This LPG sensor is referred as CA[8]. After all experiments (VOCs measurements) were completed with CA[8] sensor, the LPG was immersed into NH_3_ solution for 2 h to remove the CA[8] molecules. The LPG was then dried and immersed into CA[4] solution (the procedure was the same as for CA[8]). LPG modified with CA[4] is referred as the CA[4] sensor.

The transmission spectra of the LPG during CA[8] and CA[4] infusion were measured, along with monitoring the intensity change at a particular wavelength located on the edge of one the resonance bands. The process was terminated when the changes in the central wavelengths of the resonance bands and in the intensity were saturated.

### 2.4. Volatile Organic Compounds Measurement

The LPG modified with the CA[8] and CA[4] was tested for VOCs sensitivity in the laboratory and during a paint leakage test simulation as follows.

The LPG was fixed in a closed cell and the solution of the VOC of interest (benzene, toluene, acetone and chloroform), of volume of 10, 30, 50, 100 μL (according to the saturation level), was injected in the cell (standard Petri dish with volume of 100 cm^3^) and then was left until complete evaporation. The cell was opened shortly before adding each volume. The LPG was kept straight and taut to avoid any bending which can affect its performance. The relation between the injected volume and the VOC concentration in the Petri dish for each compound tested is shown in Table 1.

The temperature and relative humidity (RH) were simultaneously recorded as both are interfering factors (the LPG itself is sensitive to temperature, while SiO_2_ NPs thin film coating causes the sensitivity to RH). A ‘One Wire’ sensor with precision of 0.5 °C and 0.6% was used to control temperature and RH during the all experiments (iButton^®^ Hygrochron Temperature/Humidity Logger, part number DS1923, from Maxim Integrated^TM^, High Wycombe, UK). 

**Figure 2:**
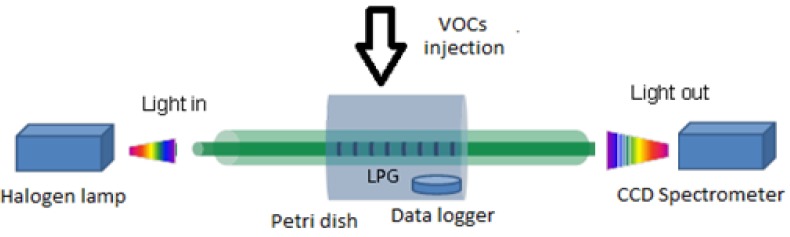
Scheme for VOCs sensitivity experiments.

The scheme used to assess the sensitivity of the LPG to VOCs is shown in Figure 2.

### 2.5. Materials Leakage Test

**Figure 3:**
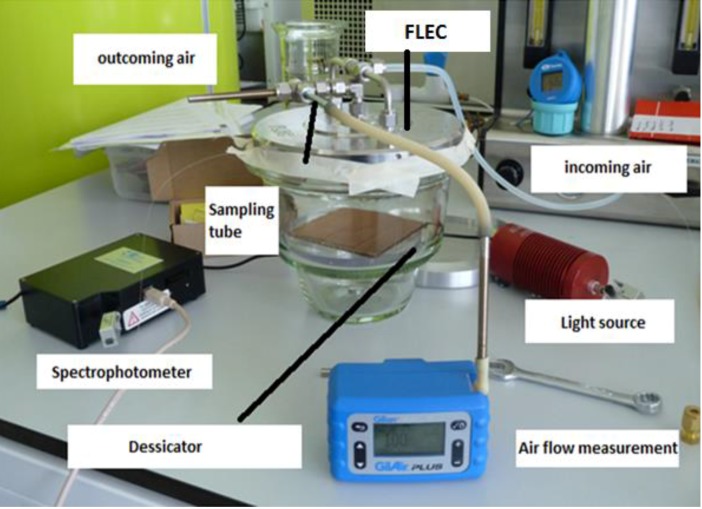
The photograph of measurement set-up used for the paint leakage test [28].

The simulation of the ISO standard test involved exposure of the LPG sensor to the mixture of VOCs emitted from paints within a chamber consisting of a desiccator base and a stainless steel emission cell, as shown in Figure 3. The LPG was fixed in a glass desiccator (with estimated volume of 7.5 L) for 4.5 h; a piece of cardboard of surface area 100 cm^2^, freshly painted by commercially available paints sold for domestic use, was placed inside the desiccator. The paint source was Plasti-kote project paint, 143S Antique gold (further referred as spray paint) and Wickes Master universal multi-surface primer (can paint).

TS were recorded every 1 min during all experiments. The central wavelengths of the attenuation bands were obtained using Spectrum Interrogation Routine software [43] and Origin was used for further data analysis. The temperature and relative humidity were kept at the same level during the experiments and simultaneously measured using OneWire device. 

A constant airflow was maintained during the whole experiment. The incoming air was controlled to be a 1:1 mixture of dry and humid air (initial RH in the cell was measured around 60%). An emission cell, a stainless steel enclosure designed for the testing of emissions of VOCs from materials, (FLEC^®^, Chematec, Roskilde, Denmark) was placed on the top of the desiccator base. Air provided to the device enters the enclosure from the perimeter and exits centrally where there is the facility to sample the air to determine the presence of VOCs. The FLEC^®^ is further described in the international standard ISO 16000-10, which concerns determination of the emission of VOCs from construction products. 

The air was constantly leaving the measuring system through an air sampling tube on the outlet. Sorbent tubes (packed with Tenax TA, Sigma-Aldrich, Dorset, UK) were used to collect the mixture of VOCs in the air. The air flow rate was 100 mL per minute and sample was collected for 2.5, 5 and 10 min with the final total volumes of 0.25, 0.5 and 1 L. The samples of volume 0.25 L were used as follows.

The air samples leaking from both paints were evaluated with the use of TD/GC/MS (thermal desorption/gas chromatography/mass spectrometry). The VOCs were identified according to peak area and expected retention time according to a NIST-library (version 2.0, 25 June 2008) with the similarity coefficient ca. 980 (maximum 1000) [44]. The thermal desorption technique was used to desorb VOCs from the tubes and a DB5 gas chromatography column and a mass quadropole spectrometer was used for the VOCs analysis. D8-toluene (C6D5CD3) (IS), retention time (RT) = 15.48 min was used as internal standard for GC-MS analysis. Its solution in methanol was injected to all tubes prior to analysis. Details of this type of analysis method can be found in ISO 16000-6 [45].

## 3. Results

### 3.1. Deposition of the Sensitive Film

**Figure 4:**
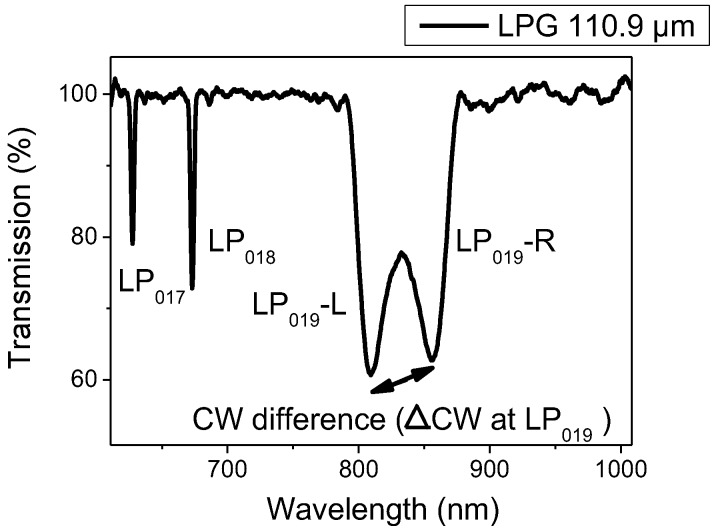
Transmission spectrum of an uncoated LPG with period of 110.9 µm measured in air, with attenuation bands at 670 nm, 800 and 850 nm corresponding to the LP_018_ and LP_019_ cladding modes respectively (the dual resonance was observed for the LP_019_ cladding mode denoted as LP_019_-L (800 nm) and LP_019_-R (850 nm)); CW, central wavelength.

The transmission spectrum of an unmodified LPG with period of 110.9 µm is shown in Figure 4. Attenuation bands in the region of 800 and 850 nm correspond to the LP_019_ cladding mode and operate at the PMTP while the other ones (region of 625 and 670 nm) corresponds to lower cladding modes and are less sensitive for this LPG period. LPG-R showed higher sensitivity than LPG-L and for this reason was used for the further analysis.

The cladding modes associated with the attenuation bands have been calculated through numerical modelling using the phase matching equitation and weakly guided approximation to determine the effective refractive indices of the core and cladding modes and the known values of the central wavelengths and the grating period [46].

**Figure 5:**
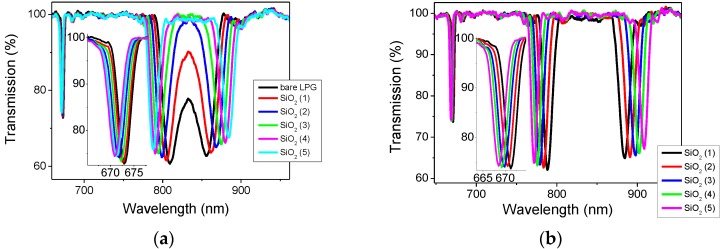
Transmission spectra of LPG with period of 110.9 µm: (**a**) taken in air before (**black**) and after the deposition of 1st (**red**), 2nd (**blue**), 3rd (**green**), 4th (magenta) and 5th layer of silica nanoparticles (cyan) and (**b**) taken in solution after the deposition of 1st (black), 2nd (**red**), 3rd (**blue**), 4th (**green**) and 5th layer of silica nanoparticles.

The LPG was coated with 5 layers of SiO_2_ nanoparticles and the changes in transmission spectrum were monitored in air and in solution, Figure 5a,b respectively. According to the results published in [15], which followed the same fabrication procedure, the thickness of the coating is 250 nm.

**Figure 6:**
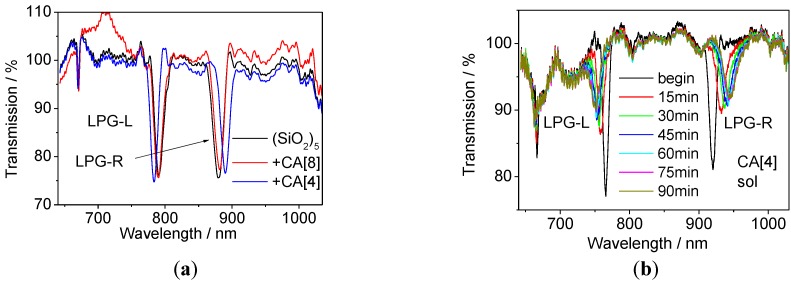
(**a**) TS after (SiO_2_)_5_ NPs deposition (black) with infused CA[8] (**red**) or CA[4] (**blue**) and (**b**) TS of LPG at the infusion of CA[8] into (PAH/SiO_2_)_8_ film deposited over LPG in solution at different time intervals.

In order to endow the sensor with sensitivity to VOCs, the LPG was initially modified by applying 5 layers of SiO_2_ NPs. The observed change in the transmission spectrum is shown in Figure 5 and it is in a good agreement with previous reports [15,25]. Comparison of the TS of the LPG measured after its immersion into CA[8] and CA[4] solutions is shown in Figure 6a. The infusion of CA[4] caused a larger shift of the central wavelengths (ΔCW at LP_019_ = 17.96 nm) than CA[8], where only a small change was observed (2.75 nm). This effect can be explained by the size of calixarene molecules. The smaller CA[4] was deposited more effectively in to the pores of the SiO_2_ mesoporous thin film, while larger CA[8] couldn’t penetrate effectively.

The evolution of the TS of the LPG when immersed in the CA[4] solution is shown in Figure 6b. The largest shift of the central wavelength as well as the absolute transmittance change at the central wavelength were observed in the first 15 min and small or no changes were observed after an hour. These results suggest that infusion of the CA[4] into the SiO_2_ mesoporous thin film saturates after 60 min.

### 3.2. VOCs Measurement

**Figure 7:**
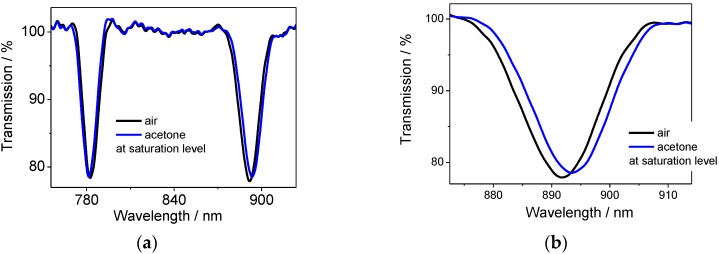
Transmission spectrum of (**a**) CA[4] sensor and (**b**) LPG-R in detail in air (**black**) and exposed to high concentration of acetone (close to the saturation level) (**blue**).

A measurable response to VOCs was observed for LPG modified with both CA[4] and CA[8] in less than 30 s (Figure 7), even when the volume of the injected solution was 10 µL, corresponding approximately to vapour concentrations of 23,000 ppm vol. for toluene, 30,000 ppm vol. for chloroform, 25,400 ppm vol. for benzene and 33,000 ppm vol. for acetone. 

With increasing amount of VOC injected the sensor response saturated at 23,000 ppm vol. for toluene, 152,000 ppm vol. for chloroform, 125,000 ppm vol. for benzene and 298,000 ppm vol. for acetone (these values are close to the saturation levels based on the boiling point). The response of the transmission spectra towards different VOCs are shown in Figures S1 and S2. As an example, acetone vapour at the concentration close to the saturation point (~298,000 ppm) induced the change in the central wavelengths difference of 2.65 nm for CA[4] sensor.

The characterization of the response of the LPG to VOCs showed that the optical fibre can respond to the high concentrations of a range of different VOCs. The results from the exposure of the sensor to individual VOCs are summarized in Table 2.

### 3.3. Material Leakage

The CA[8] sensor was exposed to the mixture of VOCs that leaked from the spray paint on the cardboard in the desiccator. Changes in transmission spectrum were observed 5 min after the painted cardboard was installed. The shift of the central wavelength continued for a further 25 min and then the changes saturated. The LPG responded immediately to opening the desiccator to remove the painted sample with the rapid change of the central wavelength observed. The final transmission spectrum was recorded three minutes after the removal of the painted sample (the desiccator was shortly opened to remove the sample and then closed again). The final spectrum was similar to that recorded prior to exposure to the VOCs, indicating good reversibility of the sensor response (Figures S3 and S6a).

**Figure 8:**
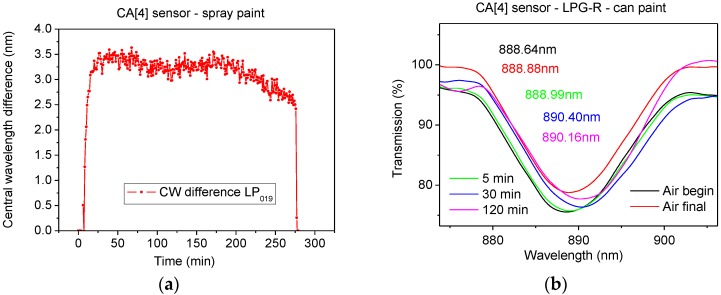
(**a**) Dynamic change of CA[4] sensor during the spray paint experiment and (**b**) CA[4] sensor—LPG-R—can paint experiment—TS—initial one (**black**), final one (**red**) and 5 min (**green**), 30 min (**blue**) and 120 min (**pink**) after the paint placement.

The CA[4] sensor was exposed to the same paint (new freshly painted sample was prepared) under the same conditions and similar results were obtained in comparison to the CA[8] sensor (Figure S4). The dynamic response of the CA[4] sensor is shown in Figure 8a. 

The CA[4] sensor was finally exposed to the can paint sample. The transmission spectra are shown in Figure 8b and Figure S5 and the dynamic response is shown in Figure S6b. A slower initial response of 0.35 nm (LPG-R) was observed in comparison to 2.22 nm shift of LPG-R observed after 5 min after the exposure to the can paint and spray paint sample respectively.

The experiments showed that the response of CA[4] sensor to the spray paint was larger than that exhibited by the CA[8] sensor, and that the response of LPG-R was larger than that of LPG-L for both sensors. This finding is in agreement with experiments exposing the sensors to individual VOCs. The CA[4] sensor responds differently to the two types of paints, most likely because of their different VOCs composition. A less pronounced response of LPG CA[4] sensor was observed mainly during the first 30 min during the experiment with the can paint. These observations support the hypothesis of the different response to the different VOCs that was found during the experiments exposing the sensors to individual VOCs. The central wavelengths of the resonance bands for selected transmission spectra are shown in Table 3.

**Figure 9:**
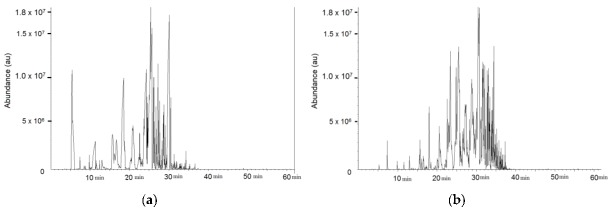
Results from GC-MS (**a**) spray paint (**b**) can paint (*x*-axis represents retention time, *y*-axis abundance).

The GC-MS analysis showed the different chemical compositions of the two paints used in this work. The results are shown in Figure 9. More than 250 different compounds were identified in both paints. This finding indicates that the VOCs with higher molecular weight are present in the can paint. Although it is difficult to obtain an exact list of the chemicals present because the individual peaks overlap, which causes biases in chemical detection, some of the VOCs could be classified. The following VOCs (5 with the highest abundance) were identified for each paint: spray—*n*-octane, *o*-xylene, *n*-nonane, Propan-2-ol, *n*-decane; can—*o*-xylene, *n*-decane, *n*-nonane, *n*-undecane, etylbenzene.

## 4. Discussion

The shift in the central wavelength difference induced by the different concentration levels of the tested VOCs is summarized in Figure 8.

**Figure 10:**
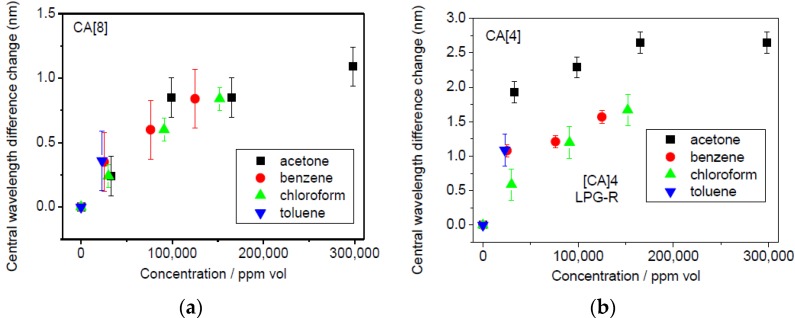
(**a**) CA[8] and (**b**) CA[4] Sensor’s response related to towards selected VOCs at different concentration levels (the error bars represent the standard deviation).

The CA[8] sensor exhibited a smaller response than CA[4] almost in all cases, which is most likely a result of the lower amount of CA[8] infused into the SiO_2_ thin film. The biggest shift was observed for acetone, the lowest for chloroform for CA[4] sensor. No differences were observed between individual VOCs for the CA[8] sensor, Figure 10. 

The response time was 1 min or less in all experiments, taken when the sensor reached 90% of the total central wavelength shift. 

It is expected that the sensor will respond to lower concentrations, as the sensor was observed to react to the smallest dose tested.

The theoretical limit of detection was stated at 2211 ppm vol. for acetone, 3057 ppm vol. for benzene, 4382 ppm vol. for chloroform and 2743 ppm vol. for toluene. The Δ CW_019_ difference of CA[4] sensor was used for the LOD calculation to enable the comparison of the sensor’s sensitivity to individual VOCs. The recalculation had been done for 10 µL injected volume. The concentration corresponding to 10 µL of the analyte was divided by the absolute shift of the central wavelength (in nm) and multiplied by 0.13 (nm) as the resolution of the spectrometer. The standard deviation across experiments where the sensor was exposed to individual VOCs ranged from 0.08 to 0.23 nm, suggesting similar values for the experimental and theoretical LOD.

The nearly same LOD to acetone, benzene and toluene and 1.5 times larger LOD to chloroform was obtained. This effect is probably caused by the smaller size of chloroform molecules, which leads to less interaction between them and the calixarene film. It should be noted that stable experimental conditions could not be attained because the chamber was not hermetically sealed and no validated measuring technique was used to measure the VOC concentration simultaneously. 

The different responses of the sensors to spray paint and to paint in a can could be explained as follows. There were higher concentrations of aromatic compounds in the spray paint, and higher concentrations of aliphatic compounds in the can paint. This conclusion correlates with the higher response of the optical fibre sensor to spray paint, because calixarene coated LPGs have previously shown higher sensitivity to aromatic VOCs than to aliphatic VOCs [30].

No degradation of the functional coating has been observed throughout the experiments, likely a result of the hybrid nature of the sensitive film, which consists of organic and inorganic moieties. Moreover, reversible response was observed due to the weak and reversible chemical interaction between calixarenes and VOCs.

The experiments with the paints were undertaken in conditions which are similar to those used for tests which determine the emission of VOCs from building products and furnishing, which are covered in British standards and international ISO norms. This kind of testing of VOC emission from different materials could be a further target for the application of fibre optic sensors after optimization of the sensor performance.

The experiments also showed the potential of measuring total or selected VOCs with use of optical fibre technology, however sensitivity at low concentrations (ppb range) has not yet been achieved. 

The sensitivity achieved to date does not meet the criteria for the 8 h exposure given by Health and Safety Executive (HSE), stated as 500, 50, 2 and 1 ppm for acetone, toluene, chloroform and benzene respectively and neither the 15 min exposure limit of 1500 and 100 ppm for acetone and toluene respectively. Further work is required to optimise the sensitivity of the sensor.

## 5. Conclusions

The performance of a fibre-optic long period grating with a nano-scale functional coating of SiO_2_ NPs with infused CA[8] or CA[4] for VOCs response has been explored. The sensors were exposed to individual VOCs and their mixtures evaporating from the freshly painted surfaces.

The ability to sense VOCs using functionalized LPGs has been demonstrated, but further work is required to improve the sensitivity, by optimization of the grating period and coating thickness. Based on the experiments presented here, and with improved calibration, it is anticipated that a limit of detection lower than of 100 s of ppm could be achieved. When the response in individual VOCs was investigated, an initial response of the sensor was observed in less than 30 s, and the sensors exhibited difference responses to individual VOCs and mixtures. Two different sizes of calixarene molecules were tested, with CA[4] exhibiting the largest response.

Although the sensor cannot discriminate between individual VOCs, a different response was observed to two different commercial paint products, suggesting that the sensor can distinguish between the different mixtures. The different responses can be explained by the presence of lower amounts of VOCs in the can paint and by the smaller response of the sensor to larger molecules. This hypothesis will be tested in future work. 

The proposed sensor benefits from easy fabrication and low cost fabrication process. The flexibility of the deposition method allows also re-use of the same fibre after cleaning. The response of the sensor was affected by the timescales for the evaporation of the VOC, but was found to be less than 1 min, enabling in situ, repeatable and real time measurement of TVOCs.

We can conclude that the LPG reacts to high concentrations of different types of individual VOCs as well as to mixtures evaporating from the freshly painted samples however the more careful control of the conditions during the lab experiments, requiring use of a hermetically sealed chamber with controllable temperature and RH and simultaneous measurement of validated techniques are needed to allow the generation of calibration curves.

Optimized operation of the sensor would require the selection of grating period, grating strength and optical thickness of the film such that the resonance band lies at the PMPT, while the thickness of the coating also ensures that the device is operating at the mode transition region [46]. An LPG operating in this was demonstrated in [26]. While there are a number of theoretical treatments of coated LPGs [47], a significant limitation in their use for predicating the optimal parameters lies in the accuracy with which the optical fibre and coating material parameters are known, which can create significant discrepancies in the predictions of the model, particularly for coupling to higher order modes at the PMTP.

It has been shown that the influence of environmental interfering parameters, such as temperature, can be corrected by using an array of appropriately configured sensors that exhibit different responses to the analyte and to the interfering parameters [48].

## Figures and Tables

**Table 1:** Relation between the injected volume and the concentration of the volatile organic compound (VOC).

VOC	Acetone	Benzene	Chloroform	Toluene
Volume (µL)	Concentration (ppm)			
10	26,012	19,806	23,679	17,978
30	78,038	59,420	71,037	n/a
50	130,064	99,034	118,396	n/a
100	260,128	n/a	n/a	n/a

**Table 2:** Sensitivity of CA[4] and CA[8] to individual VOCs.

Slope of the Calibration Curve [nm/ppm]		
Type of Calixarene	CA[4]	CA[8]
VOC	Δ CW_019_ Difference	
Acetone	5.9 × 10^−5^	1.0 × 10^−5^
benzene	4.3 × 10^−5^	1.8 × 10^−5^
chloroform	3.0 × 10^−5^	0.7 × 10^−5^
Toluene	4.7 × 10^−5^	2.0 × 10^−5^

**Table 3:** Absolute shift of the central wavelengths difference during the paint experiments.

Paint	CA[8] Spray	CA[4] Spray	CA[4] Can
Time/min	Δ CW_019_ Difference (nm)		
0	0	0	0
5	0.96	3.57	0.60
30	3.25	4.53	2.62
120	3.13	4.03	2.63
final	0.35	0.12	0.13

## Supplementary Materials

The following are available online at www.mdpi.com/1424-8220/17/2/205/s1, Figure S1: TS of the LPG with infused CA[4] exhibited to acetone (a) LPG-L; (b) LPG-R, benzene; (c) LPG-L; (d) LPG-R, chloroform; (e) LPG-L, f) LPG-R and toluene g) LPG-L, (h) LPG-R, Figure S2: TS of the LPG with infused CA[8] exhibited to acetone (a) LPG-L, benzene (b) LPG-L; (c) LPG-R, chloroform (d) LPG-L; (e) LPG-R and toluene (f) LPG-L; (g) LPG-R, Figure S5: CA[8] sensor—spray paint experiment—TS (a) LPG-L and (b) LPG-R—initial one (black), final one (red) and 5 min (green), 30 min (blue) and 120 min (pink) after the paint placement; (c) dynamic change of the central wavelength during the whole experiment, Figure S6: CA[4] sensor—spray paint experiment—TS (a) LPG-L and (b) LPG-R—initial one (black), final one (red) and 5 min (green), 30 min (blue) and 120 min (pink) after the paint placement, Figure S7: CA[4] sensor—can paint experiment—TS LPG-L—initial one (black), final one (red) and 5 min (green), 30 min (blue) and 120 min (pink) after the paint placement, Figure S8: (a) Dynamic change of a) CA[8] sensor during the spray paint experiment and (b) CA[4] sensor during the can paint experiment—LPG-L (red) and LPG-R (black).
