# Supplementary material for: Volatile Organic Compounds Sensing Using Optical Fibre Long Period Grating with Mesoporous Nano-Scale Coating
Source: Sensors (Basel). 2017 Feb 8;17(2):205. doi: 10.3390/s17020205 (PMC5335930; doi:10.3390/s17020205)
Supplement: Supplementary file 1 [file sensors-17-00205-s001.pdf]

Supplementary Materials: Volatile Organic Compounds Sensing Using Optical Fibre Long Period Grating with Mesoporous Nano-Scale Coating

Jiri Hromadka, Sergiy Korposh, Matthew Partridge, Stephen W. James, Frank Davis, Derrick Crump and Ralph P. Tatam

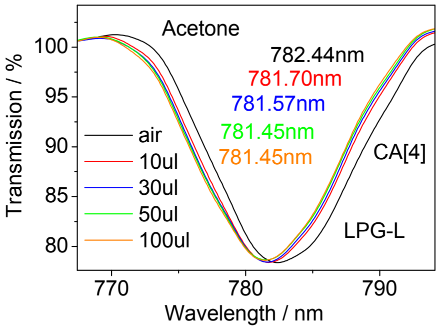

(**a**)

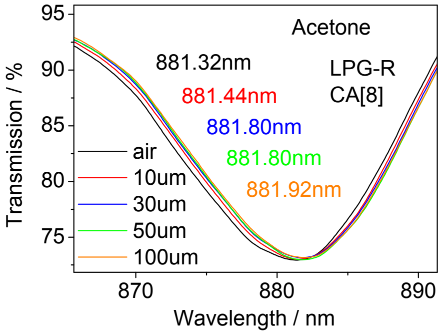

(**b**)

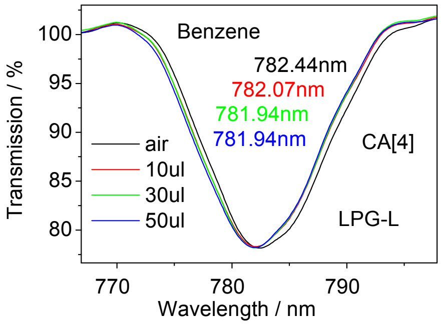

(**c**)

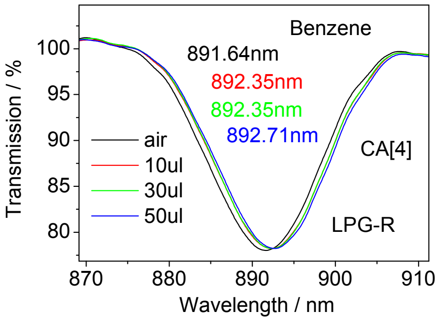

(**d**)

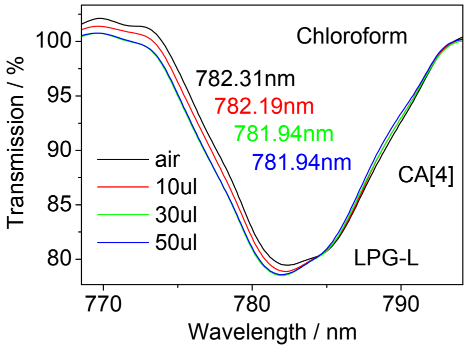

(**e**)

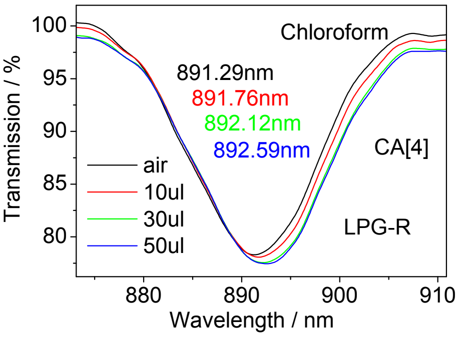

(**f**)

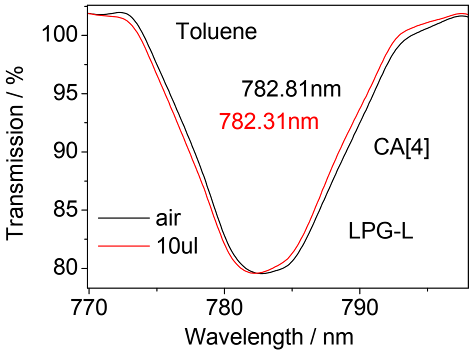

(**g**)

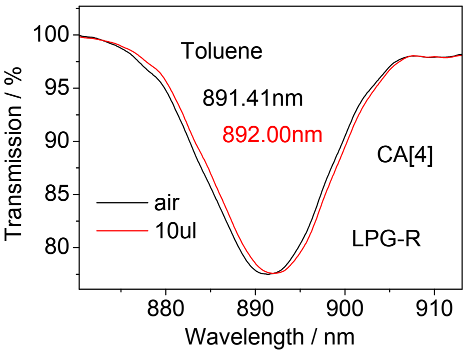

(**h**)

Figure S1. TS of the LPG with infused CA[4] exhibited to acetone (**a**) LPG-L; (**b**) LPG-R; benzene (**c**) LPG-L; (**d**) LPG-R, chloroform (**e**) LPG-L; (**f**) LPG-R and toluene (**g**) LPG-L; (**h**) LPG-R.

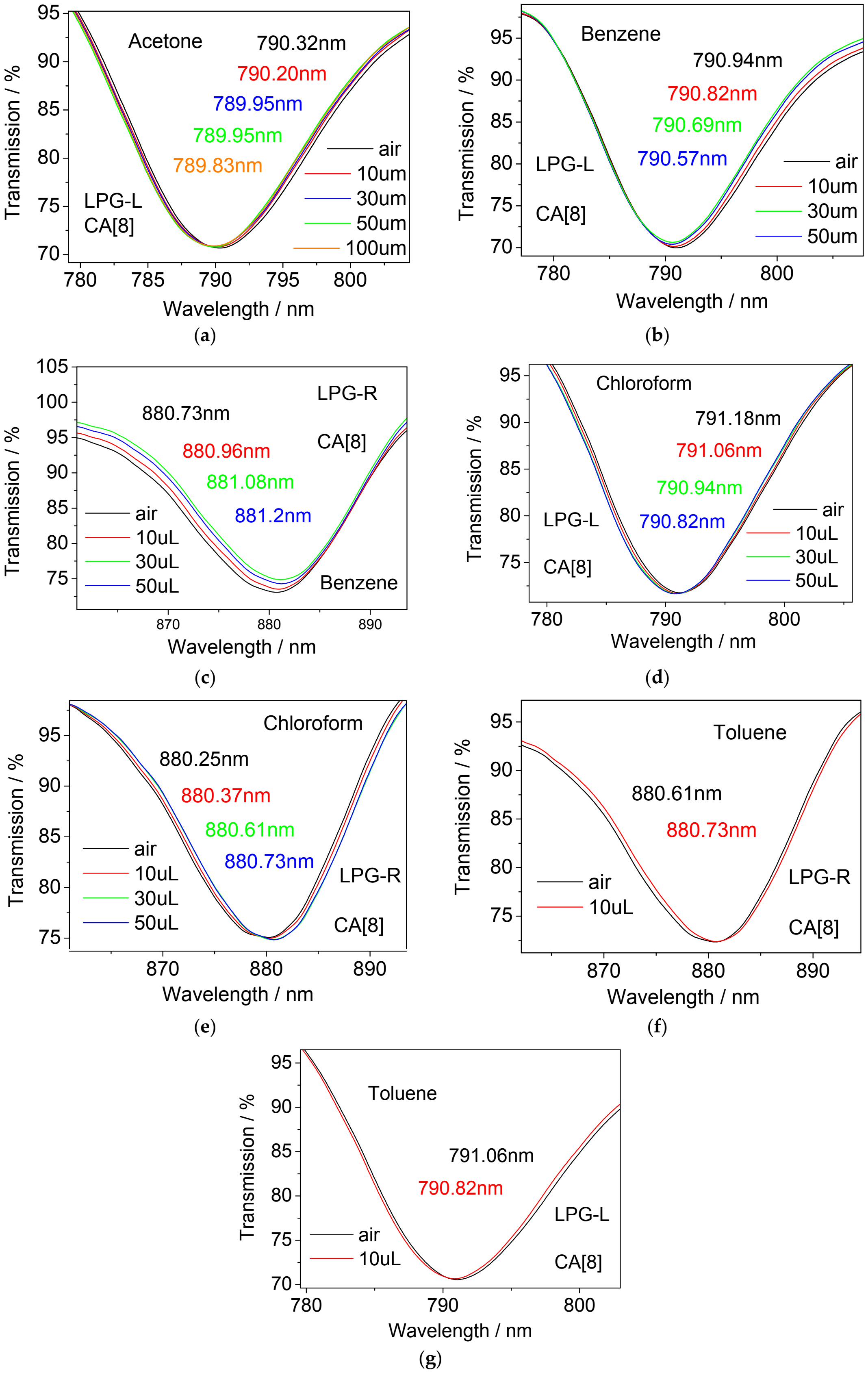

Figure S2. TS of the LPG with infused CA[8] exhibited to acetone (**a**) LPG-L; benzene (**b**) LPG-L; (**c**) LPG-R; chloroform (**d**) LPG-L; (**e**) LPG-R and toluene (**f**) LPG-L; (**g**) LPG-R.

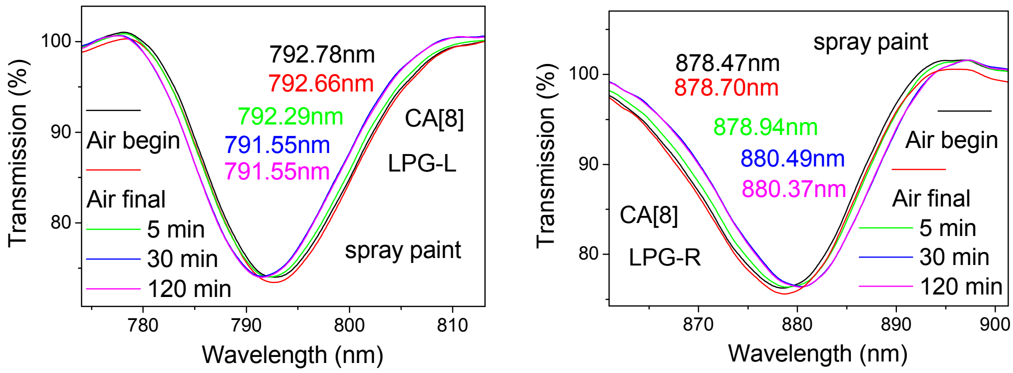

(**a**) (**b**)

Figure S3. CA[8] sensor—spray paint experiment—TS (**a**) LPG-L and (**b**) LPG-R—initial one (black), final one (**red**) and 5 min (**green**), 30 min (**blue**) and 120 min (**pink**) after the paint placement; (**c**) dynamic change of the central wavelength during the whole experiment.

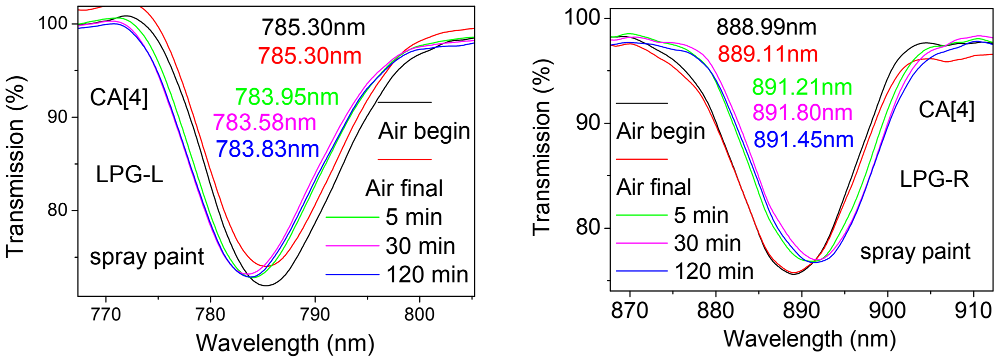

(**a**) (**b**)

Figure S4. CA[4] sensor—spray paint experiment—TS (**a**) LPG-L and (**b**) LPG-R—initial one (**black**), final one (**red**) and 5 min (**green**), 30 min (**blue**) and 120 min (**pink**) after the paint placement.

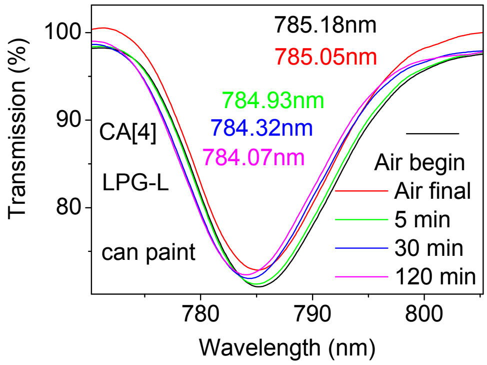

Figure S5. CA[4] sensor—can paint experiment—TS LPG-L—initial one (**black**), final one (**red**) and 5 min (**green**), 30 min (**blue**) and 120 min (**pink**) after the paint placement.

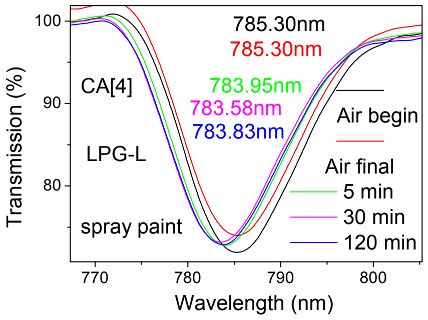

(**a**)

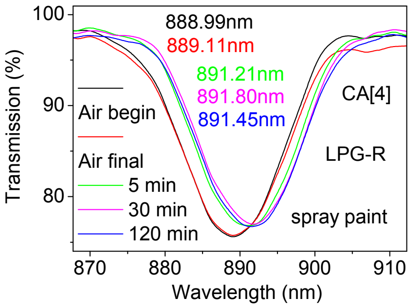

(**b**)

Figure S6. CA[4] sensor—spray paint experiment—TS (**a**) LPG-L and (**b**) LPG-R—initial one (**black**), final one (**red**) and 5 min (**green**), 30 min (**blue**) and 120 min (**pink**) after the paint placement.

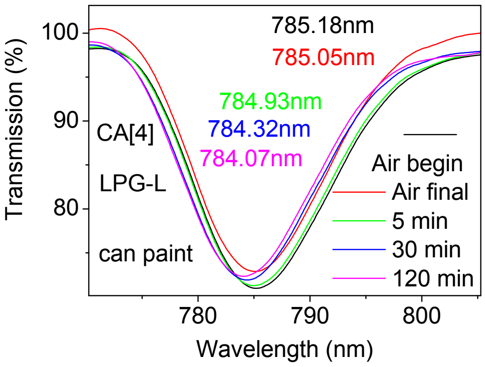

Figure S7. CA[4] sensor—can paint experiment—TS LPG-L—initial one (**black**), final one (**red**) and 5 min (**green**), 30 min (**blue**) and 120 min (**pink**) after the paint placement.

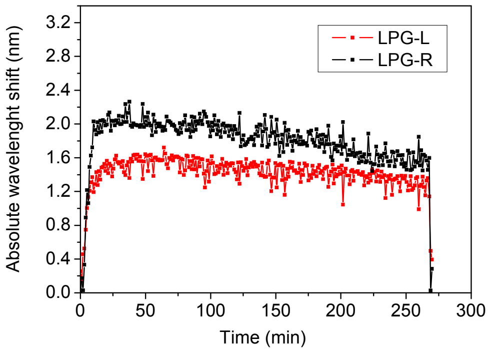

(**a**)

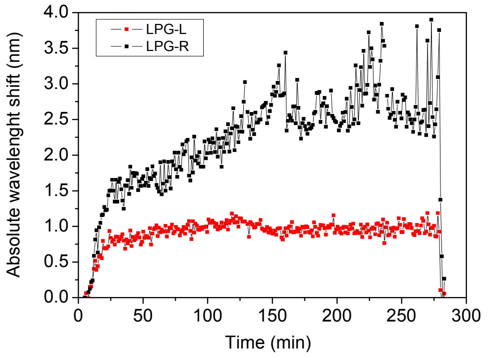

(**b**)

Figure S8. (**a**) Dynamic change of (**a**) CA[8] sensor during the spray paint experiment and (**b**) CA[4] sensor during the can paint experiment—LPG-L (**red**) and LPG-R (**black**).
